# The dual role of cyclin C connects stress regulated gene expression to mitochondrial dynamics

**DOI:** 10.15698/mic2014.10.169

**Published:** 2014-09-14

**Authors:** Randy Strich, Katrina F. Cooper

**Affiliations:** 1 Department of Molecular Biology, Rowan University School of Osteopathic Medicine, Stratford NJ, USA.

**Keywords:** cyclin C, transcription, mediator, MAPK signal transduction pathway, mitochondria, programmed cell death

## Abstract

Following exposure to cytotoxic agents, cellular damage is first recognized by a variety of sensor mechanisms. Thenceforth, the damage signal is transduced to the nucleus to install the correct gene expression program including the induction of genes whose products either detoxify destructive compounds or repair the damage they cause. Next, the stress signal is disseminated throughout the cell to effect the appropriate changes at organelles including the mitochondria. The mitochondria represent an important signaling platform for the stress response. An initial stress response of the mitochondria is extensive fragmentation. If the damage is prodigious, the mitochondria fragment (fission) and lose their outer membrane integrity leading to the release of pro-apoptotic factors necessary for programmed cell death (PCD) execution. As this complex biological process contains many moving parts, it must be exquisitely coordinated as the ultimate decision is life or death. The conserved C-type cyclin plays an important role in executing this molecular Rubicon by coupling changes in gene expression to mitochondrial fission and PCD. Cyclin C, along with its cyclin dependent kinase partner Cdk8, associates with the RNA polymerase holoenzyme to regulate transcription. In particular, cyclin C-Cdk8 repress many stress responsive genes. To relieve this repression, cyclin C is destroyed in cells exposed to pro-oxidants and other stressors. However, prior to its destruction, cyclin C, but not Cdk8, is released from its nuclear anchor (Med13), translocates from the nucleus to the cytoplasm where it interacts with the fission machinery and is both necessary and sufficient to induce extensive mitochondria fragmentation. Furthermore, cytoplasmic cyclin C promotes PCD indicating that it mediates both mitochondrial fission and cell death pathways. This review will summarize the role cyclin C plays in regulating stress-responsive transcription. In addition, we will detail this new function mediating mitochondrial fission and PCD. Although both these roles of cyclin C are conserved, this review will concentrate on cyclin C's dual role in the budding yeast *Saccharomyces cerevisiae.*

## ROLE 1: Cyclin C IS A TRANSCRIPTION FACTOR REPRESSING STRESS-RESPONSIVE GENES

### Cyclin C-Cdk8 kinase is a part of the Mediator complex

The cyclin protein family was initially identified as promoters of cell cycle progression by binding and activating cyclin dependent kinases (Cdks). As indicated by their name, cyclins display a periodic expression pattern with their levels peaking at specific stages during mitotic cell division (reviewed in 1). However, other cyclin-Cdk kinases were subsequently discovered that regulate transcription rather than cell cycle progression 2,3. This group, cyclin C-Cdk8, cyclin H-Cdk7 and cyclin T-Cdk9 also share a commonality by associating with the RNA polymerase II machinery. Of these, cyclin C-Cdk8 share the most sequence conservation from yeast to man 4. Structural analysis revealed specific determinants that promote cyclin C-Cdk8 interaction 5,6. In addition, unlike other Cdks, Cdk8 does not require phosphorylation of the canonical T-loop for activation. Rather, the presence of an atypical acidic amino acid appears to have replaced this requirement 6.

**Figure 1:**
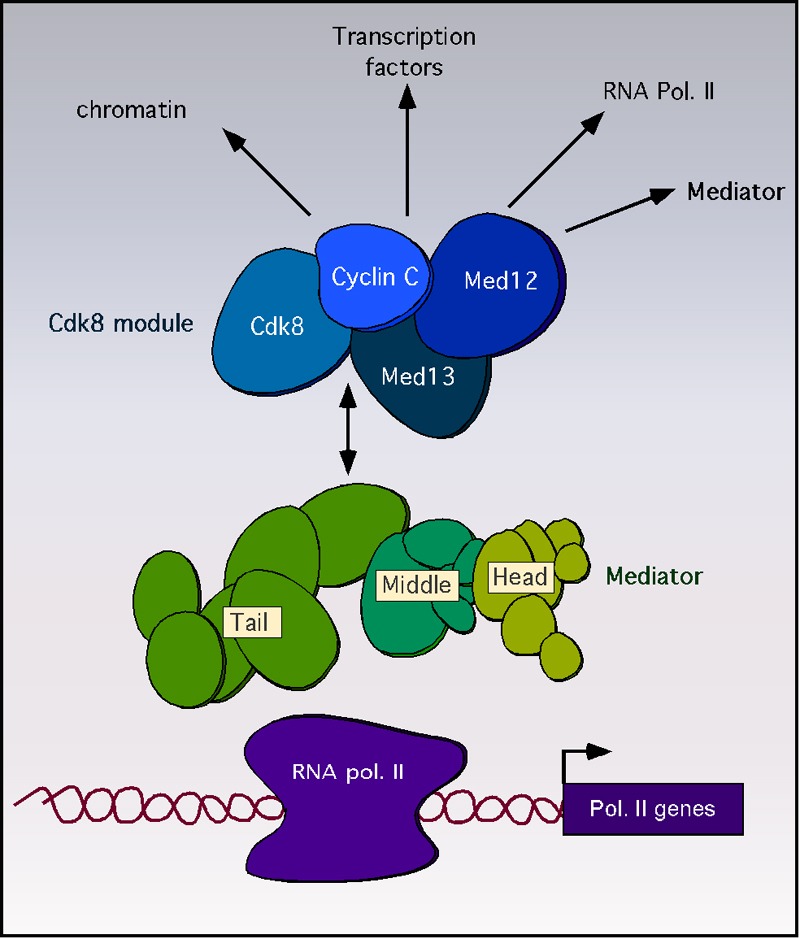
FIGURE 1: Diagram of the Mediator complex depicting the targets of Cdk8 module regulation. Cartoon of the RNA polymerase II holoenzyme bound to DNA. The core mediator complex with the tail, middle and head regions are indicated. The Cdk8 module composed of cyclin C, Cdk8, Med12 and Med13 is indicated. Reported regulatory targets of cyclin C-Cdk8p are indicated by arrows.

The cyclin C-Cdk8 kinase controls transcription though modification of the basal transcriptional machinery 7, chromatin 8,9 or transcription factors 10,11. Recruitment of cyclin C-Cdk8 to promoters occurs through the Mediator, a large complex that plays a central role in modulating RNA polymerase II activity 12,13 (and reviewed in 14). The mediator is comprised of 25-30 protein subunits that reside in four distinct domains termed head, middle, tail and the Cdk8 module (Figure 1). The Cdk8 module consists of cyclin C, Cdk8 and two additional proteins Med12 and Med13 15,16. This module is highly conserved and can be found either free 17 or associated with 12 the Mediator complex. The stoichiometry of the Cdk8 module is 1:1:1:1 and it associates with the core Mediator through the bridging function of Med13 17,18.

### The cyclin C-Cdk8 kinase primarily represses transcription of genes responding to environmental cues

Potential targets of cyclin C-Cdk8 that affect transcriptional control include other mediator components, transcription factors, chromatin and the RNA polymerase II itself (see 19 for review). Genetic studies in yeast first identified cyclin C (a.k.a. Ume3, Srb11, Ssn8) and Cdk8 (Ume5, Srb10, Ssn3) as negative transcriptional regulators of genes responding to environmental stimuli 20,21,22,23, (see 24 for review). Subsequent studies in yeast found that cyclin C-Cdk8 represses over 100 genes, many of which are considered stress response genes 25,26. Although expression profiling indicates that cyclin C-Cdk8 plays largely a negative role in transcription, there are also reports of a positive role for this factor 10,27,28. These positive and negative transcriptional regulatory roles of cyclin C-Cdk8 are dependent on specific promoter contexts (see 19,29 for recent reviews). Consistent with this observation, phenotypic studies have found that cyclin C-Cdk8 is required for processes that respond to a variety of external cues including meiotic development 30, pseudohyphal growth 11 and oxidative stress 25,31.

### The stress-activated cell wall integrity MAPK pathway relieves cyclin C-Cdk8 repression

**Figure 2:**
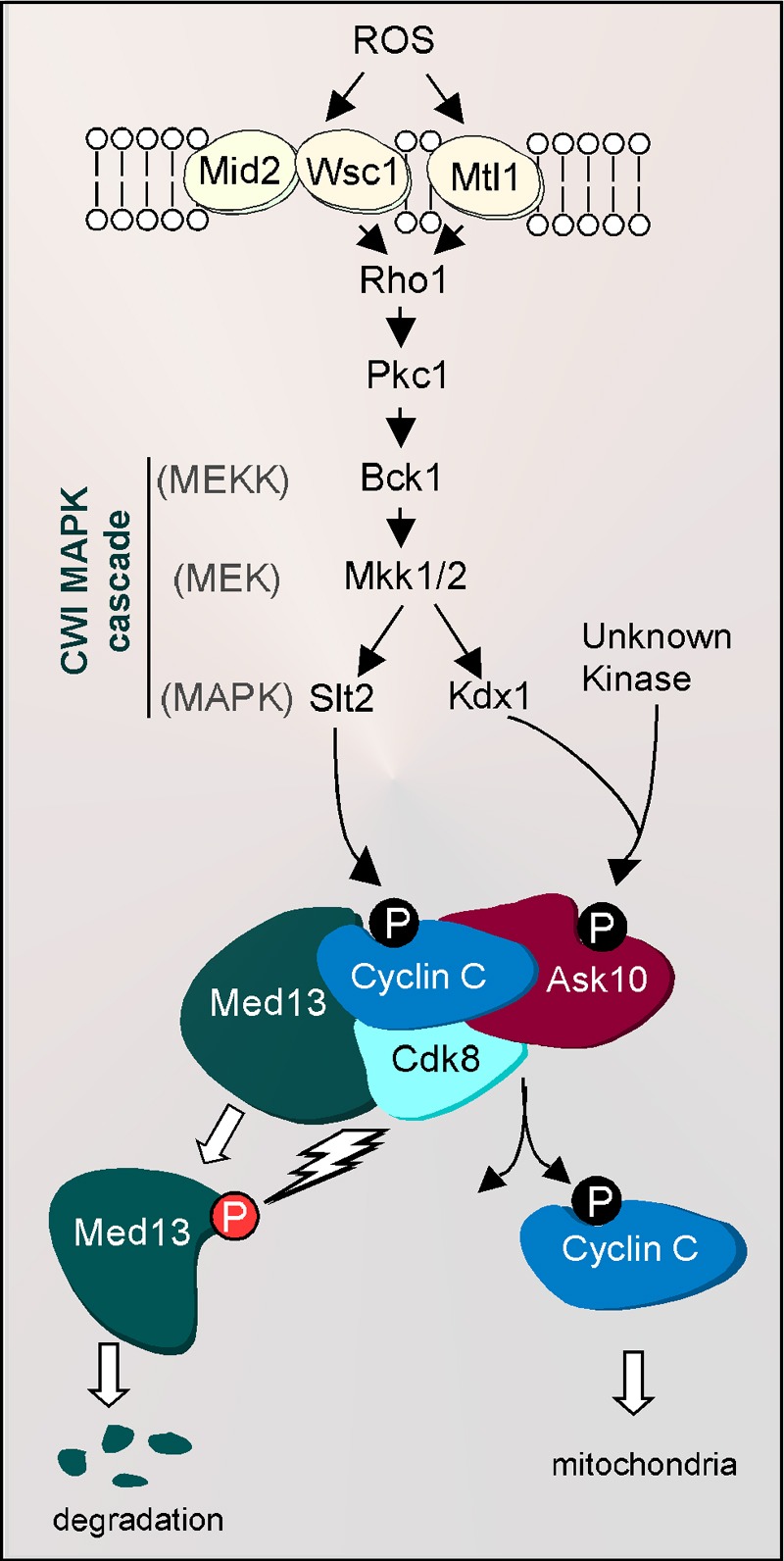
FIGURE 2: Regulation of cyclin C relocalization by the cell wall integrity pathway following H_2_O_2_ stress. H_2_O_2_-induced damage is recognized by the cell wall sensors Wsc1, Mid2 and Mtl1. These sensors transmit the stress signal via Rho1 to the Cell Wall Integrity (CWI) MAPK pathway resulting in the phosphorylation of Slt2 and Kdx1 (P). Activated Slt2 translocates to the nucleus and phosphorylates cyclin C at serine 266. Activated Kdx1 is also imported into the nucleus where it binds Ask10, which permits Ask10 phosphorylation by an unknown kinase. CWI activation leads to cyclin C translocation to cytoplasmic where it associates with the fission machinery to induce mitochondrial fission. Following fission, cyclin C is ultimately degraded via ubiquitin-mediated proteolysis. In addition, cyclin C-Cdk8 activity is required for ubiquitin-mediated Med13 proteolysis, an event that is required for cyclin C’s release from the mediator complex.

The other side of the coin with respect to cyclin C-Cdk8 repression is how this activity is removed to allow gene induction. Unlike cyclins that regulate the cell cycle, cyclin C levels do not vary significantly during the cell cycle in yeast or human cells 32,33. However, in yeast, cyclin C-Cdk8 repression is relieved by cyclin destruction 32,34 through a Not4 ubiquitin ligase-dependent process 31. In yeast, the cell wall integrity (CWI) signal transduction pathway responds to a variety of stresses including ROS 35, heat shock 36 and defects in CWI. The CWI pathway senses stress via cell-surface sensors (Wsc1-3, Mid2 and Mtl1, reviewed in 37) that transmit the signal to a small G protein Rho1 (reviewed in 38). Activated Rho1 stimulates protein kinase C (Pkc1, 39,40) and the MAPK module composed of the MEK kinase Bck1, the redundant MEKs Mkk1 and Mkk2 41, and the MAPK Slt2/Mpk1 42, or its pseudokinase paralog Kdx1/Mlp1 (43 and see Figure 2).

To affect transcription, the CWI pathway stimulates two well-characterized transcriptional activators Rlm1 and the heterodimeric factor Swi4-Swi6 (also termed SBF). Slt2 phosphorylates Rlm1 within its transcriptional activation domain to stimulate DNA binding 43,44. Interestingly, although Slt2 phosphorylates Swi6 45, a non-catalytic role for this kinase and Kdx1 in SBF-dependent activation has been described 46. A non-catalytic role for transcriptional regulation is also observed during transcription elongation as well as initiation 47. Importantly, both Slt2 and Kdx1 are activated by phosphorylation on their respective T-loop domains by Mkk1/Mkk2 46. In addition to stimulating transcription factors involved in stress gene induction, the CWI pathway is also responsible for mediating cyclin C destruction. For the cyclin C destruction pathway, oxidative stress is sensed by a complex combination of cell wall receptors (Wsc1, Mid2, Mtl1) whose activities are dictated by the level of oxidative damage 48. For example, under low oxidative stress conditions, Mtl1, and either Wsc1 or Mid2, are required jointly to transmit the oxidative stress signal to initiate cyclin C destruction. However, when exposed to elevated oxidative stress, the activity of only one of these sensor groups is necessary to destroy cyclin C. In addition, *N*-glycosylation is important for Mtl1 function, as mutating the receptor residue (Asn42) or an enzyme required for synthesis of *N*-acetylglucosamine (Gfa1) reduces sensor activity 48. These results provide a mechanism by which the cell is able to discern high- from low-level ROS damage to mediate cyclin C destruction.

Similar to activation of other transcription factors regulated by this pathway, the route the stress signal takes from Pkc1p to cyclin C is bifurcated at the MAP kinase step. The Slt2 MAPK directly phosphorylates cyclin C at Ser266 49. Eliminating this phosphorylation site prevents cyclin C proteolysis while a phosphomimetic mutation enhances its destruction kinetics. Conversely, the pseudokinase Kdx1 interacts with Ask10, a previously identified cyclin C associating factor 50. Ask10 is required for efficient cyclin C destruction and is phosphorylated in response to H_2_O_2_ 49,50. Interestingly, Ask10 phosphorylation requires the MEKs Mkk1 and Mkk2, the pseudokinase Kdx1, but not Slt2 49,50. Therefore, these results suggest the activity of another, unknown kinase in modifying Ask10 and controlling cyclin C destruction (Figure 2). Thus, cyclin C regulation is complex, as both Slt2 and Kdx1 are required for cyclin C destruction but do so through direct and indirect mechanisms, respectively. These findings emphasize that the molecular decision to destroy cyclin C is carefully regulated to prevent aberrant derepression of stress response genes.

## ROLE 2: Cyclin C MEDIATES STRESS-INDUCED MITOCHONDRIA HYPER-FISSION

### Stress-induced mitochondrial dynamics

**Figure 3:**
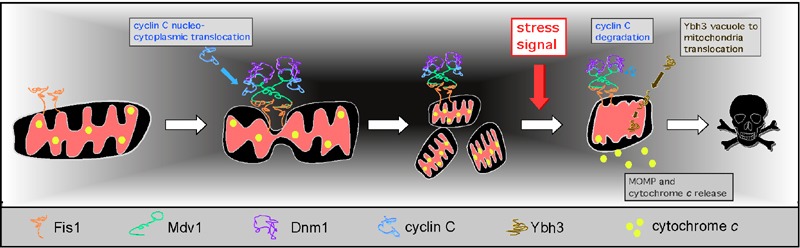
FIGURE 3: Cyclin C regulation of mitochondrial morphology and PCD following ROS stress. Upon release from the mediator complex, cyclin C enters the cytoplasm where it associates with Mdv1 promoting Mdv1-Dnm1 complex formation and extensive mitochondrial fragmentation. Thereafter, cyclin C disassociates from the fission complex and is destroyed by ubiquitin-mediated degradation. An additional stress signal, in combination with hyper-fission and Ybh3 localization to the mitochondria, is needed to complete the PCD pathway (represented by MOMP and cytochrome *c* release).

Similar to mammalian cells, yeast mitochondria serve as a signaling platform to both receive and send stress signals. Under normal growth conditions, mitochondria are usually observed in a more connected, reticular morphology (fusion) that enables maximum ATP production and the repair of mtDNA or damaged membranes by associating organelles. Conversely, mitochondrial fission allows isolation of defective organelles for removal via autophagy 51 or their efficient segregation at mitosis. However, in response to a variety of cytotoxic agents, the mitochondria undergo extensive fragmentation (Figure 3, see 52,53 for reviews). This hyper-fission is an important first step in the stress response pathway and observed in all organisms examined. Since the same machinery is utilized to execute normal mitochondrial division and hyper-fission, a stress-induced trigger for this process must be necessary.

### Mitochondrial division machinery

Mitochondrial fission requires the conserved dynamin-like GTPase Dnm1. Dnm1 is recruited to the mitochondria by the outer membrane receptor Fis1 through one of two WD-40 adaptor proteins, Mdv1 54,55 or Caf4 56. Functionally, Mdv1 acts as a nucleation factor for GTP bound Dnm1, recruiting it to the membrane via its C-terminal domain 57,58, and promoting Dnm1 to form spirals encircling the mitochondria 59. GTP hydrolysis causes ring constriction that promotes scission 60. X-ray structure analysis has revealed that Mdv1 dimerizes in an antiparallel coiled coil 61. This coiled-coil domain also promotes assembly of Dnm1 oligomers, thus is important for filament formation. Caf4 also recruits Dnm1 to the mitochondria 56,62,63,64. However, phenotypes associated with loss of Mdv1 function are more severe than in *caf4*∆ strains suggesting that Mdv1 plays a more essential role in fission 55,65.

### Mitochondrial division machinery and PCD

As described above, exposure to adverse environmental conditions shifts the delicate balance between fission and fusion dramatically toward fission 52. In yeast and higher eukaryotes, hyper-fission is proposed to be part of the process that leads to loss of mitochondrial membrane integrity and subsequent release of pro-apoptotic factors required for PCD execution. Many basic players in the PCD pathway have been conserved from yeast to humans such as caspases (reviewed in 66), a Bcl-2 family member 67 and the nucleases Nuc1p and Aif1p that are responsible for chromatin cleavage (reviewed in 68,69). However, some important mammalian PCD regulators (e.g., p53, SMAC) have not been identified to date in yeast. Consistent with a functional connection between hyper-fission and PCD, inactivating Dnm1 in yeast or its paralog in mammalian cells (Drp1) protects cells from PCD-inducing agents 70,71. However, other reports have found that hyper-fission is not required for the release of all pro-apoptotic factors 72,73 suggesting that there may not be an absolute connection between the two processes.

### Cytoplasmic localization of cyclin C directs stress-induced mitochondrial hyper-fission and programmed cell death

Several observations indicate that cyclin C directs stress-induced mitochondrial fission. First, before it is destroyed, cyclin C (but not Cdk8) translocates to the cytoplasm 31 where it associates with the mitochondria 74. This latter study found that cyclin C is both necessary and sufficient for inducing extensive mitochondrial fragmentation. A mechanism to explain the role of cyclin C in mediating stress-induced fission is suggested by co-immunoprecipitation studies revealing that cyclin C associates with the fission machinery and is required for enhanced association of Dnm1 and Mdv1 in stressed cultures 74. In addition, it was shown that cyclin C-Dnm1 association does not require mitochondrial association as the interaction was detected in *fis1*∆ mutant strains. In addition, Dnm1 forms large, non-functional aggregates in cyclin C mutants. These findings indicate that cyclin C is required for normal assembly of functional Dnm1 filaments in stressed cells.

As indicated earlier, mitochondrial fission is associated with the initial stages of PCD. If there is causation between the two events, cyclin C would be predicted to be required for normal PCD execution. This is indeed the case as yeast strains lacking cyclin C are more resistant to ROS-induced programmed cell death 75. However, ectopically inducing extensive fission by overexpressing cyclin C does not induce PCD 74 indicating that fission itself is not a commitment point for PCD execution in yeast. These findings suggest a two-step model for cyclin C-induced PCD. First, cyclin C localization to the mitochondria induces extensive fragmentation of this organelle. However, another cellular damage signal is required for the cell to commit to the cell death pathway. The nature of this signal is unknown at present. Interestingly, these results are different than those obtained with other PCD inducers that function at the mitochondria. For example, ectopically targeting p53 or bax to the mitochondria in non-stressed cells is sufficient to induce cell death 76,77. Likewise, overexpression of the yeast BH3 domain protein (Ybh3) is also sufficient to induce cell death 66. Therefore, cyclin C appears to represent a different class of regulator that is necessary and sufficient for hyper-fission but only necessary for efficient PCD.

### Med13p anchors cyclin C in the nucleus in unstressed cells

As stated above, in the nucleus, cyclin C-Cdk8 are components of a subcomplex of the mediator composed of Med12 and Med13. Recent studies have revealed that Med13 functions as an anchor protein that retains cyclin C in the nucleus in unstressed cultures 78. Deleting *MED13* allows constitutive cytoplasmic localization of cyclin C resulting in continuously fragmented mitochondria. The consequence of constant mitochondrial fragmentation is a loss of organelle function due to mtDNA deletions and a hypersensitivity to oxidative stress. To dissolve cyclin C-Med13 interaction, Med13 is subjected to ubiquitin-mediated destruction that is dependent on Cdk8 activity. In yeast strains expressing a kinase dead derivative of Cdk8, Med13 destruction is abrogated and cyclin C fails to leave the nucleus or nucleolus (Figure 2). Therefore, cyclin C release from the nucleus requires Slt2 phosphorylation and Med13 destruction, perhaps mediated by Cdk8 phosphorylation. These findings elaborate a complicated biochemical switch controlling cyclin C release that involves multiple signal transduction pathways and proteins that interact directly with cyclin C.

## CONCLUSIONS AND FUTURE PERSPECTIVES

In response to stress, the cell must sense cellular damage, transmit this signal to the nucleus to alter gene expression programs, then finally alert the remainder of the cell to the damage. In the case of cyclin C, the cell has utilized re-localization strategies to solve this problem. Following the upstream and downstream components of this regulatory system, we observe an example of how the cell has been able to integrate multiple functions into a single protein. Being a single cell organism, yeast routinely encounters cytotoxic compounds that alter membrane fluidity, generating a signal that is transduced to the nucleus (Figure 3). In the nucleus, cyclin C phosphorylation induces its release from the nucleus resulting in derepression of stress response genes through inactivation of Cdk8p. In addition, its relocalization to the mitochondria represents an intracellular signal inducing extensive remodeling of the organelle that may result in cell death. This dual role for cyclin C allows the cell to couple gene expression with organelle dynamics to produce a coordinated response. In addition, both roles for cyclin C have been conserved in human cells (our unpublished results). These observations indicate that cyclin C-dependent control of transcription and mitochondrial dynamics is a very ancient process. It is clear that as metazoans developed, additional regulatory layers were applied to both transcriptional and PCD control. However, using yeast as a model has allowed the field to peel back the years to distill the basic regulatory and mechanistic threads of these diverse processes. Such knowledge will be important not only to provide a basic understanding of how these critical events are orchestrated, but also will help identify new players in these pathways that may provide new strategies to attack diseases such as cancer. For example, the ability to manipulate cyclin C localization affects cellular sensitivity to cytotoxic agents. Therefore, only a detailed knowledge of how this system works in normal cells will allow rational designs of potential therapeutics to be realized.
